# Area, not number, dominates estimates of visual quantities

**DOI:** 10.1038/s41598-020-68593-z

**Published:** 2020-08-07

**Authors:** Sami R. Yousif, Frank C. Keil

**Affiliations:** Psychology Department, Yale University, New Haven, USA

**Keywords:** Psychology, Human behaviour

## Abstract

The study of numerical estimation collectively spans hundreds of papers and hundreds of thousands of citations. Interest in this topic hinges on one assumption: that we can approximate number independently of continuous spatial dimensions (e.g., area). Accordingly, many studies have specifically tried to demonstrate sensitivity specific to number while controlling other dimensions. However, recent work demonstrates that perceived area (based on psychophysical judgments) differs from true area (i.e., a precise pixel count). This difference raises concerns about most past studies of approximate number, by asking if they have systematically controlled for the wrong dimension(s). Building on recent findings that the percept of area may be systematically illusory, the current study examines the relation between perceived area and number. Four experiments reveal that (1) perceived area, but not mathematical area, strongly influences numerosity judgments, (2) perceived area influences perceived number but not the reverse, (3) number acuity is greatly reduced in stimuli controlled for perceived area, and (4) the ability to make area discriminations on the basis of ‘additive area’ but not mathematical area predicts number discrimination ability. Together, these findings highlight a potentially serious confound in prior work, raising new theoretical and methodological challenges for the field.

## Introduction

The ability of human adults, infants, and nonhuman animals to rapidly approximate large numbers is a cornerstone of the field of numerical cognition. This propensity purportedly relies on an evolutionary ancient system—the Approximate Number System—that serves as perceptual foundation for downstream numerical and mathematical ability^1–3^. Yet this widely accepted notion may be less plausible when considered more closely. How often would number rather than area be the most relevant cue for approximating quantity? If foraging for food, for example, would you prefer to have 100 berries, or 50 berries four times in volume? Clearly, the total volume of berries is more valuable. Despite this intuition, area approximation (as well other continuous dimensions; e.g.,^10,11^) has been vastly understudied relative to number approximation (but see^4–9^).

Despite this neglect, visual area approximation may be a fundamental ability in its own right. Recent work reveals how area perception may be illusory, suggesting that we approximate area using a visual cue other than area itself^12^. Instead, we may rely on an ‘Additive Area Heuristic’ (AAH), approximating area as the sum of objects’ dimensions rather than their product. In other words, imagine that you have a $4 \times 4$ square. To calculate that square’s area, you would simply multiple the length times the width, yielding an answer of 16. But this new perspective proposes that, instead of multiplying these dimensions, the visual system may perform a simpler calculation: adding these two dimensions together (yielding eight in this case). When ‘additive area’ is controlled, both adults and children are unable to discriminate displays that differ in true area by as much as 30%^12,13^.

**Figure 1:**
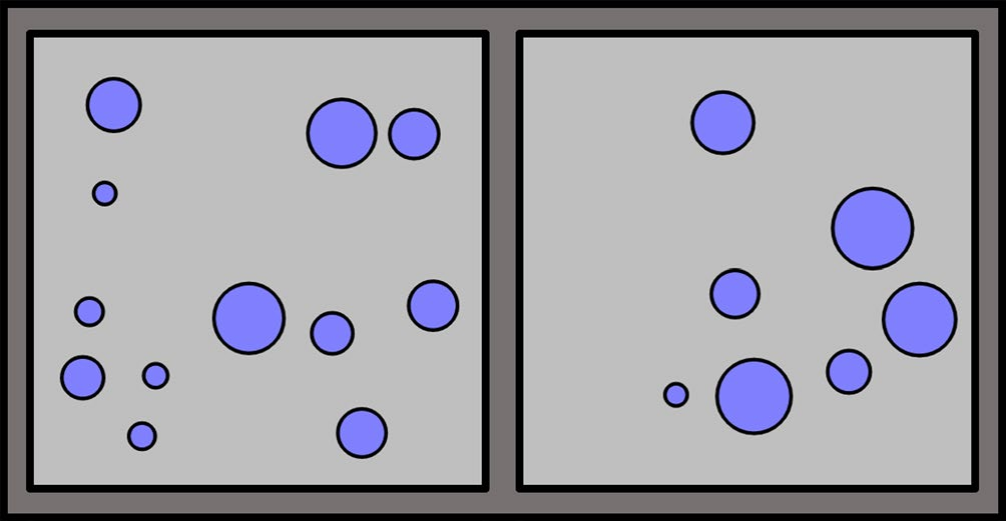
An example display for Experiments 1–4.

This model yields compelling demonstrations. For example, consider Fig. 1. Which side has more cumulative area? (If it helps, you can ask yourself which side of the display would require more ink, if printed.) Most observers report that the left side of the display is greater in cumulative area than the right, but this is not the case. In fact, the two are equal in true, mathematical area. What is different about these displays—and what seems to be responsible for this illusion—is that the left display has greater ‘additive-area’. In other words, if you were to sum the diameters of the dots in the left panel, that value would be greater than the sum of the diameters in the right display. This ‘additive-area’ estimate appears to track area estimations quite accurately, even after accounting for variables like number and contour length^12^.

The AAH raises new questions about the relation between area and number. Although numerous papers have documented bidirectional ‘congruity effects’ between area and number (in which increased area leads to an increased percept of number, and vice versa; e.g., 14–19), recent work accounting for perceived area per the AAH suggests there is no effect of numerosity on perceived area^12^. Instead, the seeming bidirectional relation may reflect a confound between perceived area and numerosity^12^. Yet it remains an open question whether area influences perceived numerosity. Prior research has often asked this question under a false premise, assuming that true, mathematical area is an accurate reflection of the percept of area (e.g.,^14–16^). Thus, the relationship between number and area remains uncertain.

In this paper, we explore congruity effects between area and number for two reasons. First: many studies making claims about relations between area and number depend on controlling area. Yet those claims may be on unstable footing when they rely on mathematical area, as they have always done. These findings should be re-examined while accounting for perceived area. Second, quantity estimation exists at the boundary of cognitive (e.g.,^2,8^) and perceptual^10^ abilities. Understanding the perceptual factors (like area estimation) contributing to quantity estimation is necessary to understand the extent to which number invokes sensation, perception, cognition, or all three. Our studies ask whether number deserves the special status that has been attributed to it (e.g., see^11^).

### Current study

Although prior work has provided clear evidence for an AAH in area perception^12,33^, the relevance of these findings to the perception of number is unknown. The present studies ask a simple, direct: could taking perceived area (discussed here as ‘additive area’) into account meaningfully challenge known findings in the literature? Perhaps even when accounting for perceived area, bidirectional interactions between area and number still exist. However, the opposite pattern would be striking, as numerous studies employing many different methodologies have generally concluded that such bidirectional interactions exist (e.g.,^14–19^).

In all of our experiments, observers viewed two displays of discs side-by-side and were asked to indicate which is greater in number (or area). An example display can be seen in Fig. 1. In the following experiments, we show that (A) perceived area (but not mathematical area) influences numerosity judgments (Experiment 1), (B) perceived area influences approximations of number but not the reverse (Experiment 2), (C) number acuity is greatly reduced in stimuli controlled for perceived area as opposed to stimuli controlled for mathematical area (Experiments 3 and 4), and (D) that the ability to make area discriminations on the basis of perceived area but not mathematical area predicts number discrimination ability (Experiment 4). Combined, these experiments raise questions about how area has been controlled in prior work, and about how area ought to be controlled in future work. The theoretical and pragmatic implications of these results—and their relation to existing work on number approximation—are discussed.

### Dissociating ‘additive-area’ and ‘mathematical area’

All of the experiments here rely on a dissociation between several correlated dimensions: namely number, mathematical area, and additive area. These dimensions can be effectively dissociated in only a few specific ways (assuming the use of simple geometric stimuli, as is by far the most common practice). For example, suppose you have a square that is $10 \times 10$. That shape has a true, mathematical area of 100 units and an additive area of 20 units. Suppose you then have two other squares, each of which are $5 \times 10$. Combined, those two shapes will have true, mathematical area of 100 units (50 each) and an additive area of 30 units (15 each). In this example, we have dissociated true area and additive area, but with the additional confound of number (the two shapes are greater in number than the single shape). It is also possible to dissociate additive area and true area without confounding number; indeed, that is one of the primary aims of our experimental design. Again, imagine that you have a square that is $10 \times 10$. That shape will have a true area of 100 and an additive area of 20. Now imagine a rectangle that is $5 \times 20$. That shape will also have an area of 100, but it will have an additive area of 25. In this example, we have dissociated additive area and true area while keeping number constant. Even for symmetrical stimuli (e.g., circles), we can dissociate additive area and true area merely by adjusting the variance in diameter. E.g., ten circles with radius 10 will have less area than five circles with radius 5 plus five circles with radius 15. Their additive areas would be the same, but that is because true, mathematical area scales exponentially with radius. In the following experiments, we exploit these mathematical realities to tease apart the dimensions of interest.

**Table 1:** The precise number, AA, and MA ratios for Experiment 1.

Number ratio	Type	AA ratio	MA ratio
**1.00**	**AA constant**	1.00	1.00
		1.00	1.05
		1.00	1.10
	**MA constant**	1.00	1.10
		1.05	1.10
		1.10	1.10
**1.15**	**AA constant**	1.15	1.05
		1.15	1.10
		1.15	1.15
	**MA constant**	1.10	1.10
		1.15	1.10
		1.20	1.10
**1.30**	**AA constant**	1.20	1.10
		1.20	1.15
		1.20	1.20
	**MA constant**	1.15	1.15
		1.20	1.15
		1.25	1.15

**Figure 2:**
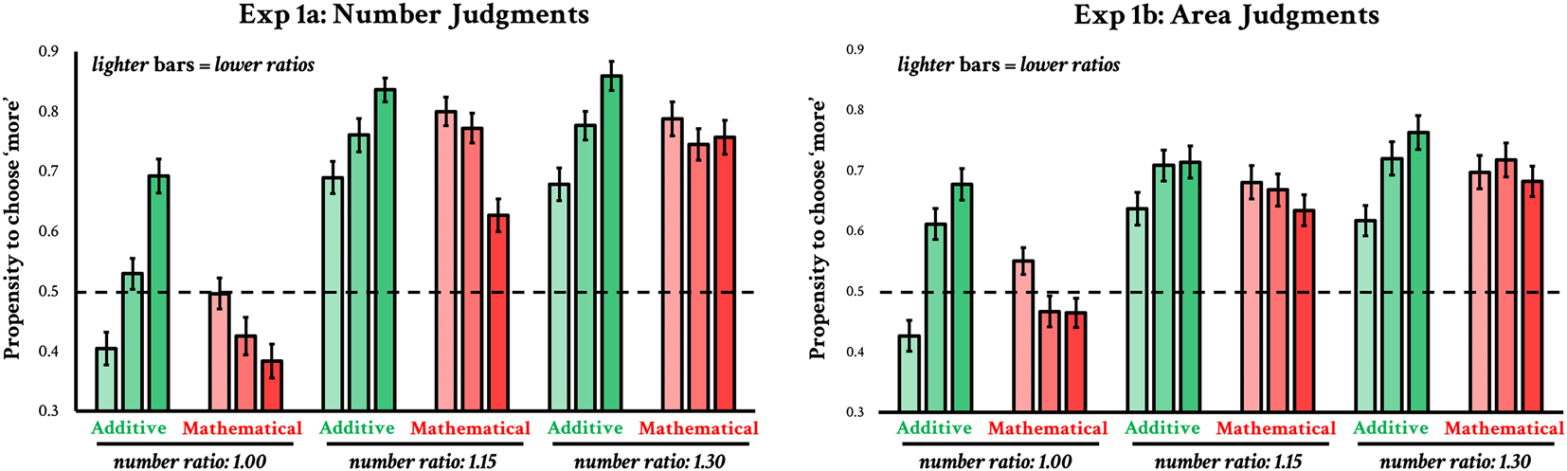
Results from Experiment 1. Results from the number discrimination task (Experiment 1a) are on the left; results from the area discrimination task (Experiment 1b) are on the right. Three number ratios are represented along the x-axis. The y-axis represents the ‘propensity to choose more’. This language is chosen because in some cases there is no objectively correct answer (in that more AA does not equal more area). Green bars represent MA-controlled sets, where AA varied in three steps. Red bars represent AA-controlled sets, where MA varied in three steps. Lighter bars represent lower ratios. E.g., for the leftmost set of green bars, the lightest bar represents the lowest AA ratio and the darkest bar represents the highest AA ratio. Error bars represent $\pm 1$ SE. The dashed line represents chance performance. Exact AA and MA ratios for each of the bars can be found in Table 1.

**Figure 3:**
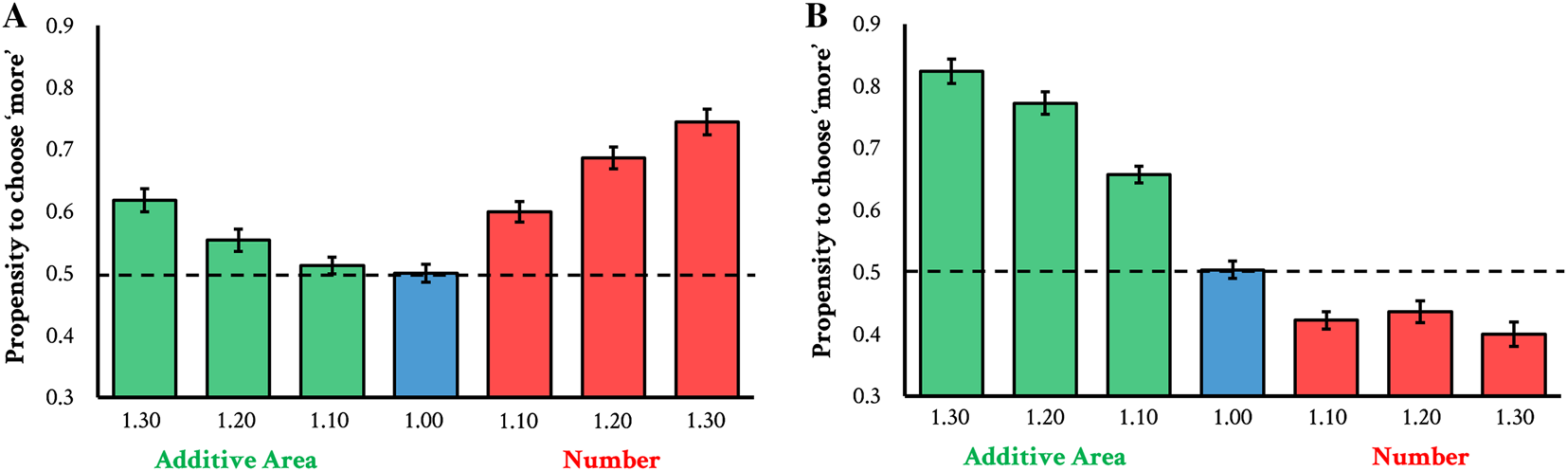
Results from number discriminations in Experiment 2a (**A**) and area discriminations in Experiment 2b (**B**). The green bars represent trials where AA varied (in a 1.1, 1.2, or 1.3 ratio) but number was held constant, while the red bars represent trials where number varied (in a 1.1, 1.3, or 1.3 ratio) while AA was held constant. The y-axis represents the propensity to choose ‘more’, whether that be more number or more area. Error bars represent $\pm 1$ SE. The dashed line represents chance performance.

## Results

### Experiment 1a: Perceived area influences perceived number

Mimicking a design in prior work^12^, we created stimuli for which additive area (AA), mathematical area (MA), and number could be manipulated independently. AA is used as a proxy for perceived area, given the prior work showing that AA captures perceived area more accurately than MA^12,13,33^. 100 observers (five excluded; see “Materials and methods”) on Amazon Mechanical Turk viewed two stimuli side-by-side and were simply asked to indicate which was greater in number (see Procedure for more information).

Three distinct number ratios were tested in this experiment: 1.00, 1.15, and 1.30. For each number ratio, there were trials of a set AA ratio and trials of a set MA ratio. Then, for a given AA and number ratio, there were three distinct MA ratios (so that MA could vary while the other two variables were held constant). Likewise, for a given MA and number ratio, there were three distinct AA ratios (so that AA could vary while the other two variables were held constant). This resulted in 72 unique trials (3 number ratios $\times$ 3 AA/MA ratios $\times$ 2 area controls [AA vs. MA] $\times$ 4 unique trials per trial type). The purpose of such a design is to fully dissociate number, AA and MA, and therefore independently assess their unique contributions to number judgments. The ranges within each dimension were carefully varied; as much as possible, we tried to ensure that different dimensions varied within a comparable range, and this was generally the case (see “Signal Clarity Theory”^20^). For additional details about the stimulus design, see Table 1 and “Materials and methods”.

If observers are properly completing the task, performance should be highest for the 1.30 ratio and roughly at chance for the 1.00 ratio. However, in addition to the main effect of number, we can independently assess the contribution of MA and AA to number judgments. For example, it is possible that for a given number ratio, more AA increases the likelihood that observers will say the stimulus has more number. The opposite may also be true: that more AA decreases the likelihood that observers will say a stimulus has more number. Finally, there may be no effect. (And all of these same possibilities apply to MA, as well.)

This study is partially motivated because, in contrast to earlier studies (e.g.,^14–18^), recent work has shown that number does not seem to influence area judgments when properly controlling for perceived area^12^. However, the question remains as to whether perceived area influences numerosity judgments. If perceived area does exert a unidirectional influence on visual number approximation, then AA but not MA should increase the likelihood that an observer will say a stimulus has more number.

The results of Experiment 1a can be seen in Fig. 2. Here, we see a main effect of numerosity, confirming that observers were able to discriminate on the basis of numerosity, $\textit{F}(2,93)=149.65$, $\textit{p}<0.001$, $\eta _{p}^{2}=0.61$. Further, increased MA generally decreased the probability that an observer would select a stimulus as more numerous $\textit{F}(2,93)=12.78$, $\textit{p}<0.001$, $\eta _{p}^{2}=0.12$. Yet, critically, having more AA increased the likelihood that observers would indicate a stimulus was more numerous, $\textit{F}(2,93)=49.08$, $\textit{p}<0.001$, $\eta _{p}^{2}=0.34$ (this trend was observed across all ratios; see Fig. 2). These results are unlikely to be due to a difference in the ratios, as they were closely matched for both AA and MA. Further, a difference in ratio would not explain the opposing effects we see for AA and MA.

We also conducted a multiple linear regression on the stimuli themselves to determine whether AA, MA, or number best predicted accuracy across the stimuli. Surprisingly, both AA ($\beta =2.40$, $\textit{p}<0.001$) and MA ($\beta =-0.98$, $\textit{p}<0.001$) were significant predictors, but number was not ($\beta =-0.25$, $\textit{p}=0.15$). Only AA predicted RTs (AA: $\beta =-388.33$, $\textit{p}=0.006$; MA: $\beta =-33.77$, $\textit{p}=0.83$; Number: $\beta =130.69$, $\textit{p}=0.17$). In other words: AA is a better predictor of both accuracy and response time in a number discrimination task. To our knowledge, such a massive overweighting of one dimension (perceived area) over another that is being explicitly probed (number) is unprecedented.

In this experiment, we did not explicitly control for cues like density or convex hull (the size of the bounding envelope of the shapes). This is lagely due to the mathematical constraints on how many dimensions can be controlled/manipulated at once. However, recent work has shown that AA explains area perception, even when explicitly controlling for these dimensions^26^. Thus, at the very least, AA is a plausible relevant dimension in this experiment.

Other dimensions, like perimeter, may also be relevant. Even though perimeter was manipulated and controlled in the original work^12^, it is possible that this dimension has an influence on number judgments. However, our core aim in this paper is not to explain area perception, but rather to document the relation between perceived area and perceived number. Nothing critical hinges on how we explain perceived area. Rather, the central issue is whether variance in perceived area explains variance in perceived number. The following experiment addresses this question.

(We also wanted to be sure that these data could not be explained by luminance, or some other idiosyncratic visual property. As such, we ran a direct replication of the results of this experiment, but with blue/green dots instead of lavender dots. i.e., one option was made up of blue shapes and the other was made up of green shapes. The blue and green colors were matched for saturation and brightness. The results replicated. The full raw data from this experiment are available on our OSF page. We thank an anonymous reviewer for this suggestion).

### Experiment 1b: Perceived area *explains* perceived number

Variance in number did not explain variance in area judgments^12^. Is the opposite true: does variance in perceived area influence number judgments? To find out, we used the exact design and stimuli from Experiment 1a. However, rather than having observers complete a number discrimination task, they completed an area discrimination task. 100 new observers (one excluded; see “Materials and methods”) participated.

The results of Experiment 1b can be seen in Fig. 2 (side-by-side with the reuslts of Experiment 1a). We first conducted a multiple linear regression on the stimuli themselves to determine whether AA, MA, or number best predicted accuracy across the stimuli. Replicating prior work^12^, only AA ($\beta =1.60$, $\textit{p}<0.001$) was a significant predictor of area judgments; number ($\beta =-0.28$, $\textit{p}=0.12$) and MA ($\beta =-0.40$, $\textit{p}=0.17$) were not.

As is evident from Fig. 2, performance across these two very different tasks (Experiments 1a and 1b) was highly correlated. We analyzed the correlation in accuracy across the 72 stimuli, asking whether stimuli that observers perceived as having more area were also perceived as having more number. The two were highly correlated $\textit{r}(70)=0.82$, $\textit{p}<0.001$. Accuracy between the experiments was also highly correlated *within* each number ratio. i.e., if we look at the correlation in ‘more’ judgments for all the stimuli with a 1.3 number ratio, any variance explained cannot be due to changes in numerosity. Nevertheless, we observe strong correlations within all three number ratios (1.00: $\textit{r}=0.80$; 1.15: $\textit{r}=0.54$; 1.30: $\textit{r}=0.61$; $\textit{p}\hbox {s}<0.01$).

Interpreted together with the prior experiment and prior work^12^, these findings suggest a unidirectional relation in which perceived area influences number, but not vice versa. In contrast to a ‘general magnitude’ account^19^, which predicts mutual positive relations between magnitudes, our findings suggest a primacy for perceived area. The following experiments test additional ‘case studies’ where AA may influence number judgments (or fail to).

### Experiment 2a: Number vs. area (number judgments)

Here, we directly pitted AA and number against each other. Borrowing from a previous design that dissociated AA and MA^12^, we manipulated both AA and number while holding the other constant. Prior studies document bi-directional congruity effects between area and number, and those studies always control MA (e.g.,^14–16^). Here, we specifically did the opposite—in order to understand whether differentially controlling for true area vs. perceived area would yield a qualitatively different pattern of results. There were seven ratios: three in which number varied (in a 1.1, 1.2, and 1.3 ratio) while AA was held constant, three in which AA varied (in a 1.1, 1.2, and 1.3 ratio) while number was held constant, and one in which both were held constant (to serve as a baseline). This resulted in 84 unique trials (7 AA/number $\times$ 12 unique trials per ratio). For additional details about the stimuli and procedure, see “Materials and methods”. Another 100 observers (three excluded; see “Materials and methods”) from Mechanical Turk were simply instructed to select the stimulus which was more numerous.

The results of Experiment 2a can be seen in Fig. 3a. Observers judged images containing more discs as more numerous ($\textit{t}(96)=11.85$, $\textit{p}<0.001$, $\textit{d}=1.20$). However, in addition, observers also judged images with greater perceived area (but equal in number) to be more numerous ($\textit{t}(96)=5.35$, $\textit{p}<0.001$, $\textit{d}=0.54$). Thus, perceived area once again appears to affect the perception of numerosity. Note that is pattern is consistent with prior work documenting congruity affects: perceived area still appears to influence number judgments (consistent with the findings of Experiment 1).

One possibility is that number estimation is automatically and irresistibly influenced by the perception of area. However, another possibility is that this pattern merely reflects a response bias. Observers—realizing there is no difference in number, but forced to make a dichotomous choice—might strategically choose the display with more of something else (in this case area). Performance on such tasks is known to be highly context-dependent, and can be distorted by subtle stimulus properties (see^21^). Experiment 2b addresses this concern.

### Experiment 2b: Number vs. area (area judgments)

If the results of the prior experiment are the product of a response bias, this bias should be bi-directional: observers tasked with an area discrimination task should choose the displays with more perceived area, but they should also choose the displays with more number when perceived area is equated. To test this bi-directional influence, we replicated Experiment 2a except that another 100 observers (three excluded; see “Materials and methods”) were asked to discriminate on the basis of area rather than number. Contrary to the response bias account, we predict that observers will not be biased to see displays with more number as having more area.

The results of Experiment 2b can be seen in Fig. 3b. Observers indicated that images greater in AA were greater in perceived area ($\textit{t}(96)=17.60$, $\textit{p}<0.001$, $\textit{d}=1.76$). However, observers were slightly below chance when selecting between displays equal in AA but differing in numerosity ($\textit{t}(96)=5.81$, $\textit{p}<0.001$, $\textit{d}=0.58$). In other words, all else equal, observers judged displays with more number as having less area—replicating the findings of experiment of recent work^12^ but in contrast to most existing work (e.g.,^14–18^).

Two conclusions emerge. First, the results of Experiment 2a cannot be explained by a response bias to simply pick the image with ‘more’ on some dimension. Indeed, observers indicated that displays with more number appeared to have less cumulative area. Second, this experiment provides converging evidence with Experiment 1 that perceived area may uni-directionally influence perceived numerosity. Although number did influence area (more number is related to less perceived area), the direction of this relationship is inconsistent with a general magnitude account and contradicts numerous papers documenting bi-directional effects (e.g.,^14–18^). In other words, the presence of *more* discs made people *less* likely to indicate that an image had more area than its counterpart.

The fact that number has an inverse effect on area judgments suggests that observers may not just be ignoring number but actively using it to make their decisions. However, unlike a general magnitude account predicts (e.g.,^14–18^), people seem to infer that more number must be indicative of less area, and thus observers are below chance. This may reflect their visual impressions, or it may reflect an overt strategy taken by participants to neglect number. One reason to suspect that this reflects an overt strategy rather than a visual impression is that there is no effect of number ratio ($\textit{p}\hbox {s}>0.05$); if number were automatically influencing area judgments, one might expect that variance in number should irresistibly affect area judgments. Regardless, area and number clearly do not possess the automatic, bidirectional, positive relationship predicted by most prior work. Perhaps future work may attempt to better understand the extent to which this pattern reflects observers’ visual impressions vs. their overt strategies.

Could these results be explained by a difference in the discriminability of AA and number? Some work has suggested that differential acuity for number and area (at given ratios) may actually explain other findings^34^. While Experiments 2a and 2b manipulated number in AA in equal ratios, their actual discriminability may have varied. Figure 3 reveals this to be true: accuracy when discriminating number (red bars in panel A) is lower than accuracy when discriminating additive area at those same ratios (green bars in panel B). But this cannot explain the relevant pattern of results. If these results were explained by differences in discriminability, then we would expect that any ratios that are generally more discriminable would have an outsize influence on the other dimensions. We can compare, e.g., 1.30 ratio for number (red bar on the far right of panel A) and the 1.10 ratio for additive area (green bar towards the middle of panel B). Accuracy is clearly higher for the 1.30 number ratio. Thus, if discriminability explains these findings, we should expect that the 1.30 number ratio is influencing area to a larger degree than the 1.10 AA ratio is influencing number—but this is not the case. In fact, the two have opposing effects: increased number appears to decrease the likelihood that a display is perceived as having more area. Nevertheless, we ran a supplemental version of this experiment using stimuli that were equated for discriminability. Results for our Experiment 2SA and 2SB can be found on our OSF page (including figures and raw data). Importantly, the results were qualitatively identical to those we report here, suggesting these results cannot be driven by a difference in the discriminability of these two dimensions.

### Experiment 3: Standard ANS task

A third experiment assessed number judgments in a classic number acuity task (in which observers discriminate sets of dots that vary in vary in number at several different numerosity ratios), while controlling for either AA or MA. Here, we predict that performance should be lower when AA is controlled. This experiment seeks not just to serve as a demonstration that AA influences number discriminations, but also to understand whether the ability to discriminate number is correlated across AA-controlled and MA-controlled trials (potentially revealing a ‘core’ number sense underlying change in superficial visual properties).

**Figure 4:**
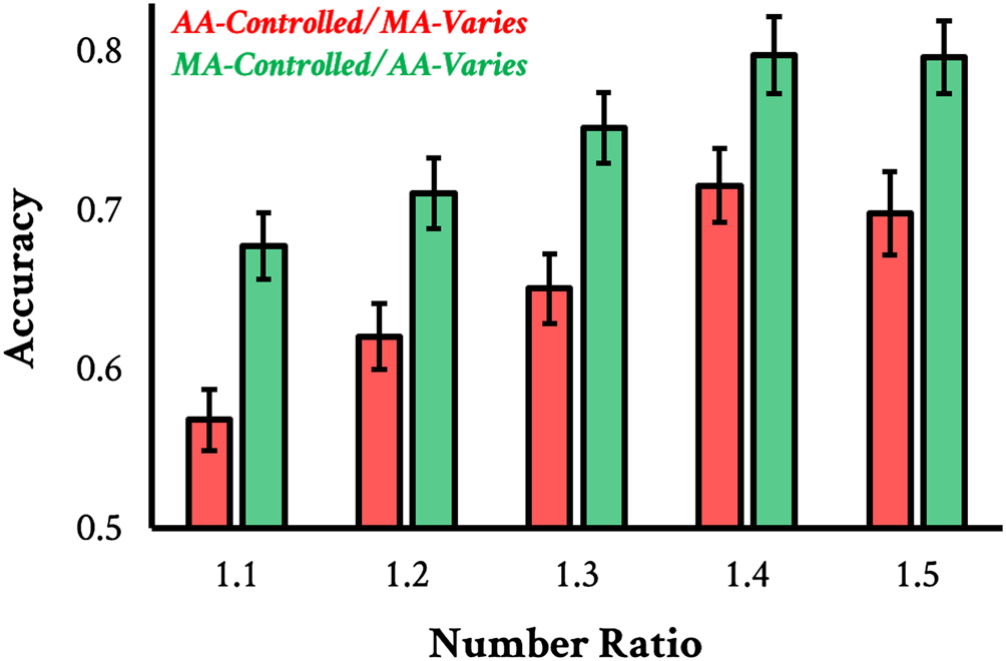
Results from Experiment 3. Five number ratios are represented along the x-axis. Green bars represent MA-controlled sets, where AA varied. Red bars represent AA-controlled sets, where MA varied in three steps. The y-axis represents accuracy for number discriminations, i.e., the proportion of time observers chose the display that was more numerous. Error bars represent $\pm 1$ SE. The x-axis corresponds to chance performance.

80 new observers from Mechanical Turk completed a number discrimination task with five distinct ratios (1.1, 1.2, 1.3, 1.4, 1.5). Critically, half the trials were AA-controlled the other half were MA-controlled. This resulted in 80 unique trials (5 number ratios $\times$ 2 area controls [AA vs. MA] $\times$ 8 unique trials per trial type). For additional details about the stimuli and procedure, see Method. The results of Experiment 3 are displayed in Fig. 4. As is evident from the figure, accuracy was indeed lower for the AA-controlled trials, $\textit{t}(78)=6.97$, $\textit{p}<0.001$, $\textit{d}=0.79$; this was independently true for each number ratio ($\hbox {ps}<0.002$). Of the 80 observers tested, 66 were as good or better at discriminating number in the MA-controlled condition (where AA varied; $\textit{p}<0.001$) Notably, performance across the two different area controls was highly correlated $\textit{r}(77)=0.69$, $\textit{p}<0.001$—about as highly as performance in each condition was to itself (MA-control: $\textit{r}=0.66$; AA-control: $\textit{r}=0.65$).

Once again, differences in perceived area dominate numerosity discrimination, accounting for substantial differences in the accuracy of number judgments. Yet, observers’ ability to discriminate number in each of these two kinds of displays was highly correlated, suggesting observers’ strategies, or their intrinsic ability to discriminate numerosity, is stable regardless of other features of the displays.

### Experiment 4: Relation between area and number approximation

Experiments 1 and 2 demonstrated how perceived area uni-directionally influences perceived numerosity. If the ability to estimate visual area is related to the ability to estimate number, then we might expect that individual differences in the ability to discriminate on the basis of AA—but not MA—would also predict the ability to discriminate perceived numerosity.

The design of Experiment 4 replicates Experiment 3, except that in addition to making number judgments, observers also made area discriminations on the same stimuli. 80 new observers (two excluded; see “Materials and methods”) from Mechanical Turk completed two counterbalanced blocks where they made either number judgments or area judgments (resulting in 160 total trials) In this way, we measured each observers’ number discrimination abilities in both AA- and MA-controlled stimuli, as well as their area discrimination ability on those same stimuli. With such a $2 \times 2$ design, we can better understand the relation between the perception of area and the perception of number. For additional details about the stimuli and procedure, see “Materials and methods”.

Consistent with the results of Experiment 3, number discrimination performance was lower for AA-controlled trials compared to MA-controlled trials, $\textit{t}(77)=3.27$, $\textit{p}<0.005$, $\textit{d}=0.37$. For the area discrimination task, performance was higher for MA-controlled trials (where AA varied) than for AA-controlled trials (where MA varied), $\textit{t}(77)=2.98$, $\textit{p}<0.005$, $\textit{d}=0.34$. Further, in the AA-controlled trials, observers were no better than chance at discriminating on the basis of MA ($\textit{t}(77)=1.64$, $\hbox {p}=0.11$, $\textit{d}=0.19$) replicating the general finding that AA but not MA governs the percept of area^12^.

**Figure 5:**
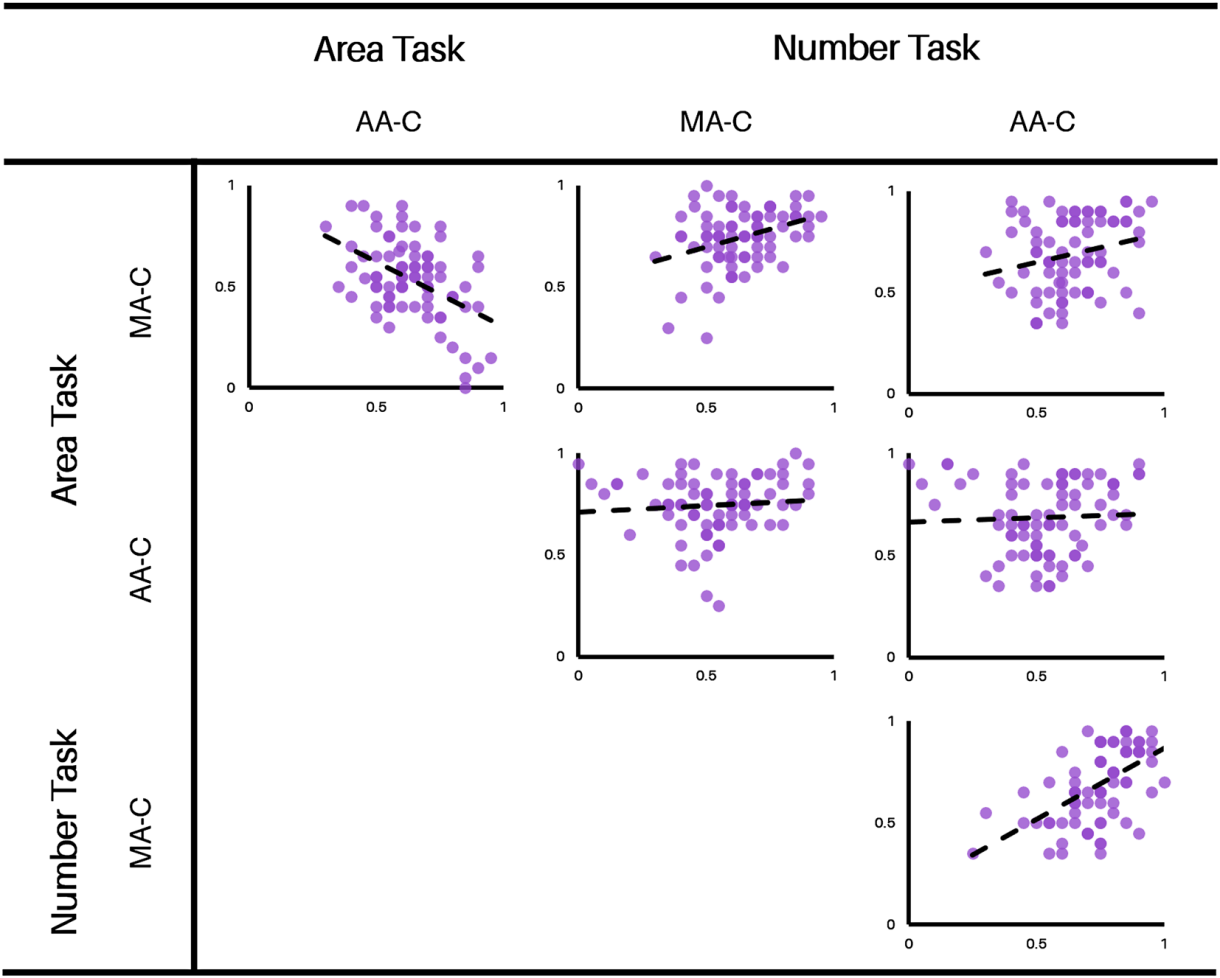
Correlations between area/number acuity on AA- and MA-controlled stimuli in Experiment 4, corresponding to Table 2. ‘AA-C’ corresponds to trials that were AA-controlled, such that MA varied. ‘MA-C’ corresponds to trials that were MA-controlled, such that AA varied. Each axis represents accuracy on the relevant task. Each dot represents an individual observer. A line of best fit is sketched for convenience.

A correlation matrix of the results of Experiment 4 can be seen in Fig. 5 and Table 2, revealing several notable patterns. Performance (i.e., accuracy) across the two area conditions was inversely correlated $\textit{r}(76)=-0.47$, $\textit{p}<0.001$ (this replicates a pattern not reported but true of prior data; 12). Performance across the two number conditions was also highly correlated, replicating the finding from Experiment 3, $\textit{r}(76)=0.56$, $\textit{p}<0.001$. Finally, performance on AA-controlled area trials (where MA varied) was not correlated with number acuity in either number condition—even when participants were making judgments on the exact same stimuli ($\hbox {rs}<0.09$, $\hbox {ps}>0.48$). However, performance on MA-controlled area trials (where AA varied) predicted numerosity performance in both number conditions, even when observers were making judgments on a different set of stimuli ($\textit{r}=0.23$, $\textit{p}<0.04$; $\textit{r}=0.35$, $\hbox {p}=0.002$). In short, visual area approximation on the basis of AA, but not MA, was related to visual number approximation. Overall, these results suggest that (a) visual area approximation is indeed driven by AA, (b) visual area approximation and visual number approximation are not fully dissociable, and (c) the ability to visually discriminate numerosity may have arisen from a more primitive ability to discriminate area.

**Table 2:** Pearson correlations of individual accuracies between area/number acuity on AA- and MA-controlled stimuli in Experiment 4, corresponding to Fig. 5.

		Area task		Number task	
		MA-C	AA-C	MA-C	AA-C
**Area task**	**MA-C**	–	*r* = − 0.473	*r* = 0.346	*r* = 0.233
		–	*p* < 0.001	*p* = 0.002	*p* = 0.040
	**AA-C**		–	*r* = 0.082	*r* = 0.048
			–	*p* = 0.478	*p* = 0.676
**Number task**	**MA-C**			–	*r* = 0.909
				–	*p* < 0.001
	**AA-C**				–
					–

‘AA-C’ corresponds to trials that were AA-controlled, such that MA varied. ‘MA-C’ corresponds to trials that were MA-controlled, such that AA varied.

**Figure 6:**
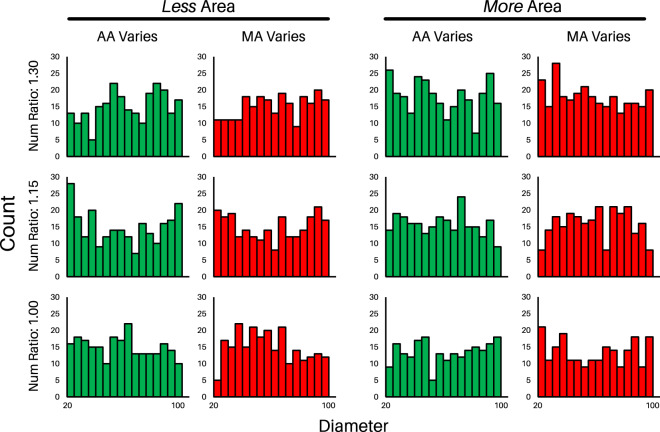
Examples of the distributions of the diameters of each dot, broken down by stimulus type, for Experiment 1. In green, you can see the diameters of the dots for the sets of trials that varied in AA. In red, you can see the diameters of the dots for the sets of trials that varied in MA. Each stimulus had a set of dots with less area (distributions shown on the left) and more area (distributions shown on the left). Along the vertical axis, these distributions are further broken down by number ratio: 1.30 on the top row, 1.15 in the middle, and 1.00 on the bottom row. Similar figures with the distributions of dots from Experiment 2–4 can be found as supplemental figures (S2 and S3) on the OSF page.

## Discussion

Is number special in visual processing? Although the field of numerical cognition is one of the largest in all of cognitive science and the ability to discriminate visual number is often thought to be the foundation of our ‘core’ mathematical competency^2^, this conclusion is not evident from first principles. In what evolutionary context would an approximate number system have been more critical for survival than approximate area or volume? Few compelling examples spring to mind.

### Theoretical implications

The issue is not whether number is special somewhere in the mind. Instead, the question is whether number is special visually—or whether, as more extreme views have suggested, it is a visual feature at all (e.g.,^10,22,23^). This question has enjoyed a wealth of discussion (e.g.,^21,24^). Yet this debate, here and elsewhere, has been plagued by the use of artificial stimuli with a seemingly immeasurable number of possible confounds. A recurring question is how one might hope to isolate numbers amidst the continuous dimensions of area, perimeter^25^, convex hull^26^, density^24^, element size^27^, cumulative diameter^28^, etc. (some of which are often anti-correlated with one another; e.g., it has been noted that some features of displays are intrinsically confounded such that displays cannot be accounted for simultaneously, as is the case for convex hull and density). And this is not even an exhaustive list of all the continuous cues that may be related to the perception of number.

The present work is not immune to the above constraints. However, a strength of our approach—distinguishing it from most prior work—is our prediction about a particular cue, AA, (rather than a collection of them) and its relation to numerosity. This prediction is based on the theoretical position that visual number estimation ought not to have been prioritized evolutionary. More consequentially, this prediction is strongly supported: perceived area (as operationalized by AA) clearly influences number, but not the other way around.

Clearly area perception is being driven by factors other than true pixel counts; that much is clear from the prior results^12^. Whether or not ‘additive-area’ is the best model to capture perceived area or not, we can firmly conclude a factor correlated with perceived area (as indicated by behavioral judgments) is influencing number discriminations (whilst number does not impact discriminations of area). Other incrementally improved models of perceived area may be presented in the future, and those results would also challenge our understanding of perceived number in the same way. In short, prior results concerning apparent numerical estimation are uninterpretable until we answer the following simple question: Do we care more about controlling true area or perceived area?

What should be said, then, about the perception of number? We have presented evidence that perceived area plays a dominant role in quantity estimation, irresistibly influencing the estimation of number. Yet we have also shown that number discrimination ability is highly correlated across very different displays (i.e., displays controlled for either AA or MA), suggesting that number estimation cannot be fully explained by perceived area (or by some superficial strategy that operates differently over different sets of stimuli). We suggest that while the human visual system is certainly able to extract number, it may not do so first and foremost and may instead reflect a subsequent level of processing. Indeed, area appears to play an outsize role in initial quantity estimation. This finding casts doubt on the strong perspective that number, like area, is a true visual feature (e.g.,^10^), while offering a new perspective on the relation between number and other continuous magnitudes.

A minor but potentially critical result of the current study is the finding that discrimination on the basis of AA predicted number discriminations, even in a separate set of stimuli that did not contain AA information. This finding, combined with the evidence that perceived area influences number but not the other way around (Experiments 1 and 2), may suggest a possible evolutionary trajectory: the ability to visually discriminate numerosities may have developed after, and be derived from, the ability to discriminate area or volume. This sequence is consistent with findings that even non-human primates have trouble discriminating number spontaneously and do so only after much training (see^1^).

### Pragmatic and experimental implications

We have characterized many of the potential theoretical implications of these findings. Whether or not one accepts this theoretical position, the pragmatic implications of these findings stand on their own. Perceived area is clearly different from true area, and all prior work has only considered true area. Here, we document several cases where controlling perceived area (AA) instead of true area (MA) substantively changes a known pattern of results and potentially challenges its core interpretation. Therefore, the present findings present a concrete and specific challenge for the field to address: what kind of area should we care about in these tasks?

Several of the findings presented here are in direct opposition to a ‘general magnitude’ view, which posits that various magnitudes share a representational structure (e.g.,^19^), and other work documenting bidirectional influences between area and number (e.g.,^14–18^). In contrast, our work shows not only that area matters more than number in quantity estimation tasks (see also^15^) but also that the influences between area and number are not bidirectional at all (.although we note that number did seem to have some influence on area judgments in Experiment 2b, it did so in a counterintuitive way that may have reflected an overt strategy on the part of the observers). There are two possibilities. (1) Magnitudes may not consistently share a representational structure, and prior findings are a result of a confound with AA. (2) These visual approximation tasks are not tapping into ‘representations’ at all. In other words, area may be prioritized in vision but may share representational space with other magnitudes in downstream processes such as memory. This dichotomy between visual perception and magnitude representations is frequently ignored, and may be one of many causes of confusion about the relation between area and number. So, again, we must ask: are there true bidirectional interactions between area and number, if these interactions only emerge when equating true but not perceived area?

The present work focuses attention on the difference between perceived area and true area. Yet, many continuous dimensions have also been discussed in prior work, including contour length^25^, density^24^, convex hull^26^, element size^27^, cumulative diameter^28^, and more. So, how should AA fit into this picture?

It is clearly not possible to perfectly isolate any of the above dimensions while controlling for all others (see^21^). As an alternative, we suggest reducing the dimensions at play by demanding principled explanations for their inclusion (as we have tried to do, by documenting the scope of the AA confound). The present work tries to accomplish this goal in two different ways. First, we take a single cue, AA, and make a prediction about that cue. We first approach area perception from first principles^12^, and then we ask whether the insights from that work apply to number approximation. This suggests that AA is likely a more important factor to control than MA. Second, while we have potentially removed the need to include MA as a dimension in these models, we have also advocated for the inclusion of another: AA seems more crucial than a dimension like contour length, as it has been directly shown to reflect visual impressions of area^12^.

Other continuous dimensions could be studied in the same way. For example, is there a single, low-dimensional cue that can capture visual estimates of density? If nothing else, one may wonder whether MA (true area) or AA (perceived area) better captures density judgments. By studying density in isolation, we might learn more about the psychophysical processes underlying density judgments, thereby allowing us to further reduce the dimensionality of existing models^27–29^.

We do not mean to suggest that studying AA is the only way forward. Indeed, many existing models have successfully taken into account numerous spatial dimensions at once^27–29^. Some of these models even incorporate dimensions (like perimeter) that, for some stimuli, are confounded with AA. Why should we care about AA if other models include similar dimensions? One reason is that the original paper ruled out dimensions like perimeter as explanations for this phenomenon^12^; therefore, AA may be uniquely implicated in area perception. Another reason is simply that no study on the basis of those models has systematically investigated how any one cue has influenced number. If they had, then those results would pose similar inferential challenges for the existing approximate number literature. In other words, we do not believe AA is the only cue that poses problems for our understanding of approximate number. It is, however, one dimension that (a) clearly tracks perception of a specific feature, and (b) influences number judgments to a larger degree.

Moving forward, the field needs work that takes both broad multidimensional approaches (as in^27–29^) as well uni-dimensional approaches (as in the present study). While our study focuses primarily on one dimension (AA) at the expense of others, other studies may ‘miss the trees for the forest’ by failing to understand any one dimension in isolation. As we show here, considering a dimension in isolation (in this case area) leads to very different results from those that have been documented before. For this reason, we believe that existing models^27–29^ may benefit from considering what cues ought to be controlled, based on independent evidence exploring each cue independently.

### Instances of apparent number dominance

Theoretical and pragmatic implications aside, there are notable cases where number appears to be preferentially processed over area. For example, in a categorization task in which stimuli varied in both number and area, people (and children, and non-human primates) spontaneously categorized ambiguous stimuli using number and not area^30^. Similarly, in a judgment task without explicit instructions (e.g., “Which has more?”), models revealed a preference for numerical information over area information^28^. Additionally, in some tasks even human infants appear to be more sensitive to changes in number than area (^31^, but see^5,6,20^).

However, the first two examples above fail to disambiguate implicit processing vs. decision-making. For example, you can imagine being asked which has more: twenty small circles, or a single massive circle? To make that decision, one would almost be forced to make an explicit choice between area vs. number. Without believing that one dimension is more important than the other, observers may arbitrarily decide that they were meant to choose one dimension or the other. In other words, when forced to reason about amount (i.e., when one is making a decision about quantity), people may rely on number—yet this reliance implies nothing about visual processing and whether people preferentially see number. Therefore, additional evidence is needed to defend these strong claims. Infants preferentially attending to changes in number could be because of a similar kind of explicit attention, or because of a simple confound with perceived area (see^12^), especially since other work suggests that area acuity may be comparable to, or superior to, number acuity early in development^4–6,20^. In addition, work documenting spontaneous categorization based on number^30^ did not control for perceived area and perceived number; it is entirely possible (if not likely, given how perceived area differs from true area; e.g.,^12^) that this result is explained by differences in the discriminability of the area vs. number changes. Indeed, recent work suggests that perceived area may influence these results^34^.

### Possible limitations

Given that our studies were conducted online, could observers’ viewing conditions influence the interpretation of our results? For example, the smallest discs in our studies were only 20 pixels in diameter, which may seem small if viewed on a mobile device like a cell phone. Could our results be distorted because some participants were simply unable to view the smallest discs? Such an influence seems unlikely (if not impossible). First, our task was designed so as to require key press inputs. To the best of our knowledge, our task could not be completed on a mobile device without attaching a separate keyboard, which is an extremely unusual situation. Second, and more importantly, our stimuli were sufficiently large and were presented in such a way that should have been clearly visible for all observers. As explained in the methods, other widely cited studies on numerosity perception use discs as small as $0.04^{\circ }$ of visual angle^35^—yet the smallest possible size of any disc in our studies (for the observer with the worst viewing conditions) would be many times this size. Finally, to be absolutely sure that viewing conditions could not have distorted our results, we separately analyzed the data based on screen size. Whether we look at the 50% of participants with the largest screen size *or* the 50% of participants with the smallest screen size, all of the patterns reported here are the same. In other words, we have no reason to believe that screen size had any effect on our results.

## Conclusion

Across numerous paradigms and stimuli configurations, one salient pattern consistently emerges: area influences number approximation but not the other way around. This is a fundamentally different pattern from what has been observed in tasks that do not control for perceived area, or AA. These findings raise pragmatic challenges for the field of numerical cognition (e.g., how should we think about and control area in displays such as these?), while offering a new theoretical perspective on the relation between number and area in vision: that number may not be so special after all.

## Materials and methods

For each experiment, observers were recruited via Amazon’s Mechanical Turk (100 each for Experiments 1, 2a, and 2b; 80 each for Experiments 3 and 4. Observers were excluded if and only if they began but did not complete the task (5 for Experiment 1a, 1 for Experiment 1b, 3 for Experiment 2a, 1 for Experiment 3, and 2 from Experiment 4). All observers provided informed consent prior to participation, and these studies were approved by the Institutional Review Board (IRB) at Yale University. All of the experiments were conducted in accordance with the IRB’s guidelines and regulations.

### Materials

All of the stimuli were generated via custom software written in Python with the PsychoPy libraries^32^. The aim was to create pairs of stimuli that varied in either AA, MA, or number while the other values were equated as much as possible. For each stimulus pair, we randomly generated an initial set of discs. In Experiment 1, the size of discs ranged from 20 pixels to 100 pixels in diameter; in Experiment 2, the discs ranged from 30 to 100 pixels in diameter; in Experiments 3 and 4, the discs ranged from 30 to 140 pixels in diameter. There was always a buffer of at least 10 pixels in between discs. Note that these pixels correspond to their size on the canvas they were originally drawn; the exact pixel values are unknowable for any given device; thus it is the relative size of discs that matters. After generating a initial set of discs, we pseudo-randomly generated a second set of objects based on a given AA/MA/number ratio (specific values varied for each experiment; see, e.g., Table 1). To get a sense of the distribution of discs diameters, see Fig. 6. In Experiments 1 and 2, the displays always had between 20 and 26 discs (the initial set always having 20). In Experiments 3 and 4, the displays always had between 10 and 30 discs (the initial set having 10 half the time, and 20 the other half of the time). Stimulus pairs were generated randomly until a pair met both the AA, MA, and number criteria, at which point that pair would be rendered another time and saved. The second stimulus always had ’more’ (whether AA, MA, or number) than the initial stimulus. For the details of how AA, true area, and number covaried, and to see the actual stimuli, see the “Stimulus Details” files on the OSF page. All discs were rendered with a thin, black border (4-pixel stroke width). The images depicted in Fig. 1 are representative of those used in these experiments.

Because of the pseudo-random nature of stimuli creation and the mathematical constraints involved in creation such stimuli, true area was never perfectly matched with the stated ratio; it could vary $\pm 1$%. That is, if the true area ratio for a given trial was 1.10, then we allowed the difference in true area to fluctuate between 1.09 and 1.11.

### Procedure

The task itself was administered online via Amazon Mechanical Turk, using custom software. On each trial, observers saw two spatially separated sets of lavender-colored discs, presented side-by-side in the center of the screen, with 50 pixels of space in between. The stimulus window was 400pixels $\times$ 400 pixels, assuming a normal browser at a standard 100% zoom. The stimuli were always counterbalanced so that an equal number containing more AA, MA, or number appeared on each side of the screen. Observers were instructed to press ‘q’ if the image on the left had more cumulative number (or area, in the relevant experiments), and ‘p’ if the image on the right had more cumulative number (or area). For the area judgment tasks (Experiments 1b, 2b, and 4), observers were told the following: “Your task is simply to indicate which set of circles has more cumulative area. In other words: if you printed the images out on a sheet of paper, which would require more total ink?” Later, they were told: “The sets of dots will sometimes vary in number, but the number of dots does not matter. Instead, you should answer only which has more area, regardless of number.” For the number judgment tasks (Experiments 1a, 2a, 3, and 4), observers were told to evaluate the number of circles. They were also given an additional warning to respond according to number regardless of area. The stimuli stayed on the screen for 700ms, but there was no time limit on responses. Between each trial, there was a 1000ms ITI. Observers completed 72 trials in Experiment 1, 84 trials in Experiment 2, 80 trials in Experiment 3, and 160 trials in Experiment 4 (again, for details on these trials, see the OSF page.) All trials were presented in a unique random order for each participant. Observers completed two representative practice trials before beginning the actual task. Because some trials in some experiments had no objectively correct answer (because either area or number did not vary), we measured accuracy as a propensity to choose ‘more’—whether that be more AA, more MA, or more number.

Because our tasks were conducted online, we cannot know the exact viewing conditions of each observer. However, we do collect information about the screen size of our participants. The median screen size in Experiment 1 was 1680 $\times$ 1050. Under these conditions (if we assume an observers sat approximately 60cm from a standard 22” monitor, which would be typical for this resolution), the average size of a disc in Experiment 1 would be about 1.5–2$^{\circ }$ of visual angle—about the size of a thumbnail viewed at arm’s length. The *smallest* disc used in any of the experiments reported here would be about a third this size, around $0.6^{\circ }$ of visual angle. Previous widely cited studies on numerosity perception have used dots as small as $0.04^{\circ }$ of visual angle^35^, one tenth this size. While some observers used smaller screens, the smallest screen size in our sample was approximately half the median. The visual angle of the smallest discs in these most extreme circumstances in our studies is still larger than in comparable studies. To gain a better sense of the size of our stimuli, we refer readers to view the stimuli themselves, available on our OSF page.

## Data Availability

Materials and data are available on our OSF page: https://osf.io/kx3sv/.
